# Anti-Inflammatory Activity of *Citrus bergamia* Derivatives: Where Do We Stand?

**DOI:** 10.3390/molecules21101273

**Published:** 2016-09-23

**Authors:** Nadia Ferlazzo, Santa Cirmi, Gioacchino Calapai, Elvira Ventura-Spagnolo, Sebastiano Gangemi, Michele Navarra

**Affiliations:** 1 Department of Chemical, Biological, Pharmaceutical and Environmental Sciences, University of Messina, Messina I-98168, Italy; nadiaferlazzo@email.it (N.F.); scirmi@unime.it (S.C.); 2 Department of Biomedical and Dental Sciences and Morphofunctional Imaging, University of Messina, Messina I-98125, Italy; gcalapai@unime.it; 3 Department of Biotechnology and Legal Medicine, University of Palermo, Palermo I-90127, Italy; elvira.ventura@unipa.it; 4 Department of Clinical and Experimental Medicine, University of Messina, Messina I-98125, Italy; gangemis@unime.it; 5 Institute of Applied Sciences and Intelligent Systems (ISASI), National Research Council (CNR), Pozzuoli I-80078, Italy

**Keywords:** bergamot, *Citrus bergamia*, inflammation, antioxidant activity, flavonoids, natural products, complementary and alternative medicines

## Abstract

Inflammatory diseases affect a large portion of the worldwide population, and chronic inflammation is a major risk factor for several dangerous pathologies. To limit the side effects of both synthetic and biological anti-inflammatory drugs, the use of herbal medicines, nutraceuticals and food supplements has increased tremendously as alternative and/or complementary medicine to treat several pathologies, including inflammation. During the last decades, the biological properties of *Citrus bergamia* (bergamot) derivatives have obtained important scientific achievements, and it has been suggested their use in a context of a multitarget pharmacological strategy. Here, we present an overview of the anti-inflammatory properties of bergamot extracts that could represent the scientific basis for develop novel and alternative strategies to improve health status and attenuate inflammatory conditions.

## 1. Introduction

Inflammation is a complex biological reaction induced by the disruption of the tissue homeostasis, occurring in response to the presence of a biological, chemical, or physical agent in the body [1]. The classical key features of inflammation are redness, warmth, swelling, and pain. Inflammation can be acute or chronic, depending on the type of stimulus and the effectiveness of the inflammatory process resolution. Acute inflammation begins quickly and lasts a few hours or a few days. It is characterized by the exudation of fluid and plasma proteins as well as leukocyte emigration (mainly neutrophils). When the immune system successfully eliminates damaging agents in acute inflammation, the reaction disappears, but if the response fails to remove them, a chronic phase occurs. Inflammation cascades can lead to the development of diseases such as chronic asthma, rheumatoid arthritis, multiple sclerosis, inflammatory bowel disease, and psoriasis. Chronic inflammation is associated with the presence of lymphocytes and macrophages, vascular proliferation, fibrosis, and tissue destruction. Experimental and clinical studies together with epidemiological observations have identified the chronic infections and inflammation as major risk factors for various types of cancer. It has been estimated that the underlying infections and inflammatory reactions are linked to 15%–20% of all cancer deaths [2].

The inflammatory response is characterized by coordinate activation of various signaling pathways that regulate expression of both pro- and anti-inflammatory mediators in cells of inflamed tissue as well as leukocytes recruited from the blood. The nuclear transcription factor κB (NF-κB) is the master regulator of the inflammatory response, that drives the activation of genes associated with the transcription of inflammatory mediators, such as interleukins, tumor necrosis factor-α (TNF-α) and prostaglandins (PGs), as well as inflammatory enzymes, like inducible nitric oxide synthase (iNOS) and cyclooxygenases (COXs). Several classes of medicines, including corticosteroids, nonsteroidal anti-inflammatory drugs (NSAIDs) and biologics drugs are used to treat the inflammatory disorders. However, they possess several side effects and the biologics ones are expensive to be used. To limit these drawbacks of both synthetic and biologic drugs, over the past three decades, the use of herbal medicines, nutraceuticals and food supplements has increased greatly as an alternative and/or complementary medicine to treat several pathologies, including inflammation [3]. Indeed, although natural products are not devoid of risk, generally they are safer than both synthetic and biologic drugs. Nowadays, about 80% of people around the world use natural products for the prevention and treatment of many diseases, mainly for their relative safety, efficacy and low cost as well as the compliance by patients. Plants have been the basis of many traditional medicines throughout the world for thousands of years, and continue to provide mankind with new remedies [4]. In this field, natural products offer great hope in the identification of bioactive molecules useful for the treatment of inflammatory diseases, as it happened for aspirin, the first discovered NSAID, which is still one of the bestselling drugs in the world. Polyphenols, such as flavonoids, lignans, phloroglucinols, quinones, stilbenes, phenylpropanoids, and diarylheptanoids, are a very important category of natural compounds able to modulate inflammatory pathways. Among them, luteolin [5,6], quercetin [7], wogonin [8,9], apigenin [6,8], fisetin [6] and baicalein [6] showed anti-inflammatory activity in vitro and in animal models.

A growing volume of evidence supports the hypothesis that the use of a phytocomplex, such as an extract, can have a greater preventive and therapeutic success than a single biomolecule, because of both additive and synergistic effects of their individual constituents [10,11,12,13]. Moreover, all phytochemicals present in whole extracts may simultaneously modulate different targets of action in human cells, leading to a mixture of pharmacological effects contributing together to improve patient’s health [14,15]. Hence, we and other researchers have started investigating the anti-inflammatory action of a number of phytocomplexes in a context of a multitarget pharmacological strategy.

In this review, we present an overview of the anti-inflammatory properties of bergamot extracts that could be the scientific basis for the development of novel and alternative strategies aimed to prevent and/or treat inflammatory conditions.

## 2. Oxidative Stress and Inflammation

Inflammation and oxidative stress are closely related pathophysiological events that are strongly linked with one another. One of them may appear before or after the other, but when one emerges the other one is most likely to follow and then both of them take part in the pathogenesis of many disorders. Nowadays, it is clear that the prolonged low-grade inflammatory process plays a central role in the pathogenesis of many chronic diseases [16]. On the other hand, epidemiological and experimental studies strongly suggest a contribution of oxidative stress in many human diseases [16]. Just as the inflammatory process can induce oxidative stress, the latter can cause inflammation through activation of multiple pathways [17,18]. The result is that both inflammation and oxidative stress are associated with a number of chronic diseases, including diabetes, hypertension, cardiovascular diseases, neurodegenerative diseases, alcoholic liver disease, chronic kidney disease, cancer, and aging [19,20,21,22].

Under pathological inflammatory conditions there may be exaggerated generation of reactive species (RS) that can diffuse out of the cells, inducing localized oxidative stress and tissue injury [23]. Moreover, the activated phagocytic cells produce large amounts of radical oxygen and reactive nitrogen species (ROS and RNS, respectively). Once generated, they can further generate other RS, leading to extensively damage. At the onset of inflammation, the infection or tissue damage is sensed by the pattern recognition receptors like toll-like receptors (TLR), NOD-like receptors (NLR), and the receptor for advanced glycation end products (RAGE). The stimulation of these receptors upon binding with specific molecules leads to the activation of transcription factors such as nuclear factor-κB (NF-κB) and activating protein-1 (AP-1), that in turn induce pro-inflammatory gene expression, exert antimicrobial functions and recruit additional immune cells [24,25]. It has been demonstrated that RS (i.e., hydrogen peroxide) can induce inflammation through activation of these same transcription factors [26,27,28].

Over the last decades, researchers have investigated on the effectiveness of antioxidants in preventing diseases like cardiovascular diseases, cancer, diabetic complications, Alzheimer’s disease, and so forth [29]. Most of the results come from in vitro studies, and clinical researches have produced controversial data, although many epidemiological data suggest that antioxidants may have a beneficial effect in many chronic diseases. However, great care should be taken in the choice of antioxidant agents and their dosage, as well as, it is most important to quantify the redox and inflammatory status to make appropriate interpretation of the findings.

## 3. Bergamot Derivatives

Known as “bergamot”, *Citrus bergamia* Risso et Poiteau is a small plant belonging to the Rutaceae family, growing spontaneously in the southern coast of Calabria region (Italy), where the particular microclimate is ideal for its cultivation [30]. Bergamot fruit is used especially for the extraction of its essential oil (BEO) from the peel (by cold pressing), while the bergamot juice (BJ), derived squeezing the endocarp of the fruits, is considered a byproduct of the BEO’s production. Finally, the scraps of bergamot fruit after both BEO extraction and BJ juicing is named “bergamot pastazzo” and is used as animal feed. BEO is typically used in the cosmetic industry, being found in the composition of many fragrances, body lotions, soaps and so on. It is also used by the food industries (for flavoring tea, beverages and typical Calabrian pastries), by the pharmaceutical industries (to absorb the unpleasant smell of medicinal products and for its antiseptic and antibacterial properties [31]) and in aromatherapy [30]. Over the last decade, some researchers have started to investigate the biological properties of bergamot derivatives, obtaining important scientific achievements. For example, BEO has been evaluated for its potential neuroprotective [32] and antiproliferative effects [11,33], while BJ has been suggested as antioxidant [34,35], anti-inflammatory [36,37,38,39], anticancer [15,40,41,42] and hypolipidemic [43,44] drug.

The chemical composition of the main *Citrus bergamia* derivatives, BEO and BJ, has been studied mostly by high performance liquid chromatography–mass spectrometry (HPLC-MS), gas chromatography–mass spectrometry (GC-MS) and gas chromatography–flame ionization detection (GC-FID) techniques, and has been reported in a number of papers [41,45,46,47,48]. BEO is composed of both volatile and non-volatile fractions that account for 93%–96% and 4%–7% of the total, respectively. The non-volatile components are responsible for phototoxic reactions at certain concentrations, due to the presence of furanocoumarins (or furocoumarins), especially bergapten [30]. The main constituents of BEO are terpens and furanocoumarins, among which limonene, linalyl acetate (terpens) and bergamottin (furanocoumarins) are the most abundant molecules. Other compounds present in fewer amounts are the terpenes linalool and γ-terpinene, the coumarins 5-geranyloxy-7-methoxycoumarin and citropten, as well as the furanocoumarin bergapten [30]. Their chemical structures are presented in Table 1. BJ is characterized by the presence of high amount of flavonoids among which naringin, neohesperidin, neoeriocitrin and eriocitrin are the most abundant, although melitidin, hesperetin and naringenin are also present [36,41,46]. The major flavonoids in BJ and their molecular structures are shown in Table 2.

A number of researchers have documented the capability of molecules present in both BEO and BJ to counteract inflammation via modulation of several pathways. For example, the pharmacological functions of coumarins, including their anti-inflammatory activity, were reviewed by Wu et al. in 2009 [49], and very recently by Srikrishna et al. [50], while the anti-inflammatory profile and the structure-activity relationship of terpenes was discussed by Souza and coworkers in a systematic review [51]. The anti-inflammatory properties of some BJ flavonoids as pure compounds have been extensively investigated and well established in several experimental models [52,53,54].

## 4. Antioxidant Properties of Bergamot Derivatives

Antioxidant compounds play an important protective role in the prevention of numerous diseases. Although the antioxidant activity of BJ has been previously addressed [55], recently we better characterized the chemical composition of a flavonoid-rich extract from BJ (BJe), and then investigated its antioxidant properties in both abiotic and cell-based assays [34,35]. First we tested the antioxidant activity of BJe using a range of tests (Folin-Ciocalteu, Reducing Power, DPPH and ORAC assays) that highlighted its great antioxidant and radical scavenging properties related to the high total phenol content [34]. Then, we focused on the cytoprotective ability of BJe against oxidants, such as hydrogen peroxide (H_2_O_2_) and (Fe_2_SO_4_)_3_, that cause oxidative cell damage [34,35]. We found that BJe prevented either H_2_O_2_ or (Fe_2_SO_4_)_3_-induced oxidative damage on the A549 cells by different mechanisms depending from the stressor agent. Indeed, the extract (25 or 50 µg/mL) reduced ROS generation and counteracted the membrane lipid peroxidation, improved mitochondrial functionality and prevented DNA-oxidative damage in A549 cells incubated with H_2_O_2_ or (Fe_2_SO_4_)_3_. We further described the chelating property of BJe in iron-exposed cells, thus blocking upstream redox activity as well as its capability to induce antioxidant catalase. Overall, our results show that BJe exerts its antioxidant properties through different complementary via: scavenging free radicals, metal ion chelation and boost of cellular antioxidant defense.

Trombetta et al. [56] evaluated the antioxidant/anti-inflammatory activity of two alcoholic flavonoid-rich extracts from bergamot peel on human vessel endothelial cells (HUVECs) exposed to the pleiotropic inflammatory cytokine TNF-α, a model of vascular oxidative stress. As hallmarks of oxidative damage in TNF-α-exposed HUVECs, the Authors monitored cell viability, intracellular levels of MDA/HNE, GSH and GSSG, and SOD activity. They showed that both extracts prevented the oxidative stress induced by TNF-α, modulated the activation of redox-sensitive transcription factors NF-κB, thus increasing the cell survival.

## 5. Anti-Inflammatory Activity of Bergamot Derivatives

The anti-inflammatory activity of bergamot derivatives has been demonstrated in both in vitro and in vivo studies. Karaca et al. [57] investigated for the anti-inflammatory activity of BEO using carrageenan-induced rat paw oedema test. The authors found that reduction in the inflammation, measured as reduction of paw volume, was 95.70% with indomethacin used as standard control, 27.56% with 0.025 mL/kg BEO, 30.77% with 0.05 mL/kg BEO and 63.39% with 0.10 mL/kg BEO (ED_50_ value of 0.079 mL/kg). Borgatti et al. [58] have analyzed the effects of a bergamot epicarp extract and the isolated cumarins on the production of IL-8 (mRNA levels and protein release) in cystic fibrosis IB3-1 and CuFi-1 cells treated with TNF-α or heat-inactivated *Pseudomonas aeruginosa*. Particularly, they showed as the most active molecules were bergapten and citropten, typically present in BEO.

Other researchers studied the potential anti-inflammatory activity of the polyphenolic fraction of BJ on normal human NCTC 2544 keratinocytes exposed to interferon-gamma (IFN-γ) or histamine (H) [59]. The juice was obtained from peeled fruits by industrial pressing and squeezing. The juice was filtered and loaded on column absorbing polyphenols. Then, polyphenolic fraction was eluted by hydroalcoholic acid solution (60:40 *v*/*v*) and dried by spray-drying. IFN-γ is an essential cytokine in amplifying inflammatory reactions, which stimulates the synthesis of chemokines, that in turn attract inflammatory cells and induces the expression of inducible intercellular adhesion molecule-1 (ICAM-1). Moreover, the over-secretion of inflammatory cytokines triggers an abnormal accumulation of extracellular matrix components such as glycosaminoglycans (GAGs) [60,61]. Keratinocytes are the major constituents of the epidermis, and in inflamed skin they up-regulate various pro-inflammatory activators, such as PGE2, NO, ROS and cytokines [62]. They are actively engaged in skin inflammatory responses by attracting leukocytes and modulating their functions. The assays performed on keratinocytes demonstrated that the bergamot extract significantly reduces ICAM-1, NO, ROS and GAG production in cells exposed to IFN-γ and H in concentration-dependent manner [59]. Moreover, these Authors observed similar results when they employed neoeriocitrin, naringin or neohesperidin, the most abundant flavonoids of the BJ. Of note, the antioxidant/anti-inflammatory effect of the wool extract was superior than that of each flavonoid used alone [59].

Nisticò and coworkers [63] evaluated the effect of a 38% bergamot polyphenolic fraction (BPF) on UVB-induced photoageing by examining inflammatory cytokine expression, telomere length/telomerase alterations and cellular viability in human immortalized HaCaT keratinocytes subjected to ultraviolet (UV) radiation. They clearly showed as the phytocomplex protected HaCaT cells against UVB-induced oxidative stress, reducing IL-1β mRNA expression and restores telomere length and activity.

Experiments performed in our research lab demonstrated that BJe is able to significantly reduce the LPS-pro-inflammatory action in THP-1 cells [36]. Indeed, we demonstrated that the extract inhibited both gene expression and secretion of LPS-induced pro-inflammatory cytokines (IL-6, IL-1β, TNF-α). Then, given the reported key role of NF-κB in inflammatory response induced by various stimuli, we also investigated its role in THP-1 cells exposed to LPS, showing that BJe was able to inhibit this important transcription factor. It is known that polyphenols could influence cellular function by acting as activators of SIRT1, a nuclear histone deacetylase involved in the inhibition of NF-κB signaling. Therefore, in a subset of experiments we also investigated the capability of BJe to modulate SIRT1, evidencing that the extract activated SIRT1, that in turn reduced the LPS-enhanced acetylation of p65 in THP-1 cells [36].

In the light of these observations, we wondered whether BJe may be beneficial against neuroinflammatory processes, such as those observed in Alzheimer’s disease, where the chronic activated glial cells amplify the neurotoxicity by secreting pro-inflammatory mediators like cytokines. Hallmarks of chronic inflamed tissues are the presence of an increased number of monocytes, as well as monocyte-derived tissue macrophages, that can be referred to microglial cells in the central nervous system (CNS) [64]. To this aim we investigated the effectiveness of BJe in THP-1 cells exposed to amyloid-beta (Aβ), an experimental model of circulating monocytes/macrophages that resemble neuroinflammatory condition [38]. Exposure of THP-1 cells to Aβ significantly induced the expression and secretion of IL-6 and IL-1β in THP-1 cells, and increased the phosphorylation of ERK 1/2 as well as p46 and p54 members of JNK family. These effects were reduced by the pre-treatment with BJe, confirming its anti-inflammatory potential. Some results show that increased cytokine expression may be due to different transcription factors, such as AP-1. Notably, in this research we observed that the increased AP-1 DNA binding activity in Aβ-treated cells was reduced by the pretreatment with BJe [38].

The anti-inflammatory activity of BJe has also been demonstrated in animal models. In an in vivo model of inflammatory bowel disease, we have shown that BJe attenuated the inflammation by dinitrobenzene sulfonic acid (DNBS) [37]. The BJe-treated mice were more resistant to DNBS-induced colitis and showed less macroscopic and histological signs of inflammatory process. Moreover, the expression of important inflammatory mediators, such as TNF-α and IL-1β, as well as the neutrophil infiltration, were reduced in the colon tissues of DNBS-injected mice as consequence of BJe treatment. We also reported that BJe reduced the increase of p-JNK expression, and inhibited the NF-κB activation in the colon of DNBS-treated animals. In addition, we found reduced expression of adhesion molecules (ICAM-1 and P-selectin), nitrotyrosine, poly (ADP ribose) synthetase (PAR) and apoptosis related proteins in DNBS-injected mice treated with BJe [37].

The beneficial effects of BJe have been also demonstrated in ileum inflammation triggered by intestinal ischemia/reperfusion (I/R) injury in mice [39]. The experimental results confirmed that BJe was able to reduce histological damage, cytokines production (TNF-α and IL-1β), ICAM-1 and P-selectin expression, myeloperoxidase (MPO) activity and oxidative stress (malondialdehyde, MDA; manganese superoxide dismutase, mnSOD and inducible nitric oxide synthase, iNOS) by a mechanism involved both NF-κB and MAP kinases (JNK and p38) pathways.

The studies on the anti-inflammatory effect of bergamot derivatives collected in this review are summarized in Table 3.

## 6. Conclusions

Despite the remarkable medical advances, inflammation remains a serious health problem. Recently, interest in natural product has led both researchers and the wellness industry to explore natural sources for alternative and complementary medicine to prevent and/or treat inflammatory diseases. Several studies on the beneficial effects of bergamot derivatives in the inflammation field have been published, increasing the attention towards this *Citrus* species as a potential source of natural antioxidant/anti-inflammatory remedies. This is the first review that collects and critically reports the literature on this topic. It may represent a scientific basis for the development of novel strategies to improve health status and attenuate inflammatory conditions.

## Figures and Tables

**Table 1:** The main constituents of BEO and their chemical structures.

BEO			
Coumarins and Furanocoumarins		Terpenes	
Name	Molecular structure	Name	Molecular structure
Bergamottin	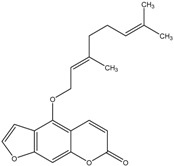	Limonene	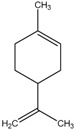
Bergapten	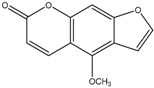	Linalool	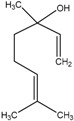
Citropten	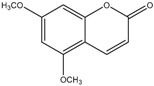	γ-Terpinene	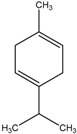
5-Geranyloxy-7-methoxycoumarin	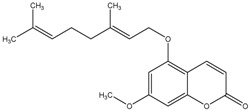	Linalyl acetate	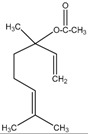

**Table 2:** The major flavonoids in BJ and their molecular structures.

BJ Flavonoids			
Name	Molecular structure	Name	Molecular structure
Naringin	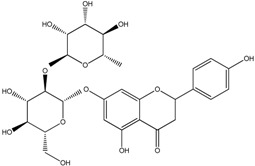	Hesperetin	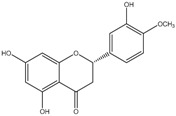
Neoeriocitrin	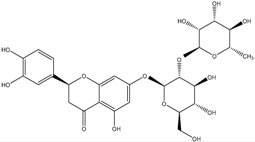	Neohesperidin	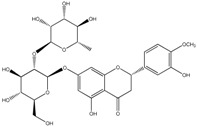
Narirutin	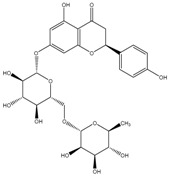	Eriocitrin	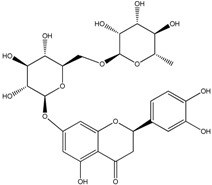
Melitidin	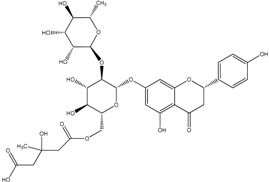	Brutieridin	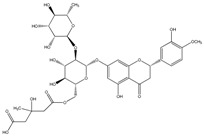

**Table 3:** Principal characteristics of studies on anti-inflammatory effect of bergamot derivatives.

Compound	Study Model	Determinations	References
BEO	Carrageenan-induced inflammation	Paw volume	Karaca et al.; 2007 [57]
Extract from bergamot peel	Endotelial oxidative stress/inflammation	MDA, 4-HNE, GSSG, GSH/GSSG, SOD	Trombetta et al.; 2010 [56]
Extract from bergamot epicarp	Cystic fibrosis	IL-8	Borgatti et al.; 2011 [58]
Extract from bergamot juice and flavonoids	Skin inflammation	ICAM-1, iNOS, NO, ROS, GAG	Graziano et al.; 2012 [59]
Extract from bergamot juice	Activated monocytes	IL-1β, IL-6, TNF-α, NF-κB, SIRT-1	Risitano et al.; 2014 [36]
Extract from bergamot juice	Colitis	IL-1β, TNF-α, MPO, nitrotyrosine, p-JNK, ICAM-1, P-selectin PAR, NF-κB, BAX, BCL-2	Impellizzeri et al.; 2014 [37]
Extract from bergamot juice	Lung oxidative stress	ROS, lipid peroxidation, mitochondrial potential, DNA damage	Ferlazzo et al.; 2015 [34]
Extract from bergamot juice	Skin photoaging	IL-1β, telomerase activity	Nisticò et al.; 2015 [63]
Extract from bergamot juice	Neuroinflammation	IL-1β, IL-6, ERK1/2, p-JNK, AP-1	Currò et al.; 2016 [38]
Extract from bergamot juice	Lung oxidative stress	ROS, lipid peroxidation, mitohcondrial potential, DNA damage, catalase	Ferlazzo et al.; 2016 [35]
Extract from bergamot juice	Intestinal ischemia/reperfusion injury	histological alterations, IL-1β, TNF-α, MDA, MnSOD, ICAM-1, P-selectin, NF-κB, iNOS, p-p38, p-JNK	Impellizzeri et al.; 2016 [39]
